# Bioelectrical Impedance Analysis of Body Composition in Male Childhood Brain Tumor Survivors

**DOI:** 10.3390/diseases12120306

**Published:** 2024-11-28

**Authors:** Alberto Romano, Fabrizio Sollazzo, Fabio Corbo, Giorgio Attinà, Stefano Mastrangelo, Simona Cordaro, Gloria Modica, Isabella Carlotta Zovatto, Riccardo Monti, Massimiliano Bianco, Palma Maurizi, Vincenzo Palmieri, Antonio Ruggiero

**Affiliations:** 1 Pediatric Oncology Unit, Fondazione Policlinico Universitario Agostino Gemelli IRCCS, 00168 Rome, Italy; corbo-fabio@virgilio.it (F.C.); giorgio.attina@policlinicogemelli.it (G.A.); stefano.mastrangelo@policlinicogemelli.it (S.M.); simona.cordaro92@gmail.com (S.C.); palma.maurizi@policlinicogemelli.it (P.M.); 2 Sports Medicine Unit, Fondazione Policlinico Universitario Agostino Gemelli IRCCS, 00168 Rome, Italy; fabrizio.sollazzo@unicatt.it (F.S.); gloria.modica01@icatt.it (G.M.); isabella.zovatto@gmail.com (I.C.Z.); riccardo.monti1@unicatt.it (R.M.); massimiliano.bianco@policlinicogemelli.it (M.B.); vincenzo.palmieri@unicatt.it (V.P.); antonio.ruggiero@unicatt.it (A.R.); 3 Department of Woman and Child Health and Public Health, Università Cattolica del Sacro Cuore, 00168 Rome, Italy

**Keywords:** childhood cancer survivor, cardiovascular risk, metabolic syndrome, cranial radiotherapy, brain tumor, sarcopenia, chemotherapy

## Abstract

**Background**. Childhood brain tumor survivors (CCSs) are at high risk of developing metabolic syndrome (MetS) and sarcopenia. To date, a tool able to predict any body composition changes or detect them early and increased adiposity (and, therefore, increased likelihood of MetS onset) is still lacking in this population. **Objective**. The objective was to analyze differences in a bioelectrical impedance analysis (BIA) of body composition between male childhood brain tumor cancer survivors and healthy controls. **Methods**. In this pilot, prospective, observational study, 14 male CCSs were compared to 14 healthy controls matched for sex and age. **Results**. CCSs showed statistically significant lower mean values in terms of their body metabolic rate (BMR), body cell mass index (BCMI), fat-free mass (FFM), skeleton muscle mass (SM), skeletal muscle mass index (SMI), and appendicular skeletal muscular mass (ASMM). CCSs also showed a statistically significantly higher mean value of resistance when compared with controls. The BMR, BCM, FFM, and ASMM were significantly correlated with total doses of carboplatin (Tau = −0.601; *p* = 0.018; Tau = −0.599, *p* = 0.025; Tau = −0.601, *p* = 0.018; Tau = −0.509, *p* = 0.045, respectively). **Conclusion**. A BIA allows for the detection of changes in body composition in survivors of childhood brain tumors, revealing either the presence of central obesity correlated with the risk of MetS or signs of sarcopenia that deserve early treatment.

## Simple Summary

Because of the treatments they have undergone, survivors of childhood brain tumors have a greater risk of cardiovascular disease and sarcopenia compared to the general population. The objective of this study was to analyze differences in a bioelectrical impedance analysis (BIA) of body composition between male childhood brain tumor cancer survivors and healthy controls and to evaluate the correlation between the BIA results and the treatments performed (chemotherapy, radiotherapy, and steroid therapy). Our analysis indicates the presence of several body composition indexes at the BIA that point towards the presence of sarcopenia in childhood brain tumor survivors, showing also a significant correlation between some of these indexes and total dose of carboplatin received.

## 1. Introduction

Pediatric brain tumors are the second most common childhood cancer after leukemia, accounting for roughly 25% of all childhood tumors [1].

It has been estimated that in the US, 15,000 people between 0 and 19 years old are diagnosed with cancer every year and that 500,000 individuals alive in the US are childhood cancer survivors [1].

The mainstay of treatment is a multimodal approach (based on surgery, chemotherapy, and radiation therapy) that raised up the overall survival to more than half of the cases [2].

Given this high rate of survival, it is mandatory to pay attention to long-term effects of cancer treatments. Among the most frequent long-term effects, the cardiovascular ones are those that more affected the morbidity and mortality of childhood cancer survivors (CCSs): death from a cardiac cause is seven times more frequent in CCSs than in the general population [3].

Due to the intensive use of chemotherapy, radiation therapy, and prolonged steroid therapy, CCSs often develop a chronic inflammatory state that leads to metabolic syndrome (MetS) and high cardiovascular risk [4,5,6]. Moreover, cranial radiotherapy in doses exceeding 30 Gy via hypothalamic–pituitary axis disfunction and GH deficiency promotes either the overproduction of leptin by adipose tissue and leptin resistance at hypothalamic receptors level or lipid and free fatty acid oxidation, insulin resistance, and changes in body composition, leading to an increased android–gynoid fat ratio and promoting the onset of dyslipidemia and central obesity [3,5,7,8,9,10,11]. Lastly, all chemotherapeutic drugs can cause the accumulation of senescent cells and reactive oxygen species that can anticipate MetS onset.

Prolonged steroid therapy also promotes the release of cytokines involved in the chronic inflammatory state, increasing the risk of diabetes mellitus, hepatic steatosis, atherosclerosis, and thrombosis [12,13]. Adipose tissue and chronic inflammation through cytokines and adipokines are positively related with total fat mass and negatively with muscle mass [14,15], as if a proinflammatory state might induce a decrease in muscle strength among obese persons. Insulin resistance [16], low levels of GH [17] and testosterone [18], and a relative state of malnutrition may contribute to the development of a condition of both muscle tissue depletion and fat mass increase known as “sarcopenic obesity” [19]. At present, both sarcopenia and elevated adiposity are known to increase the risk for developing cardiovascular disease independently because they are both associated with the occurrence of MetS and its consequences [20,21,22].

Several efforts have been made in order to detect MetS early in CCSs, focusing mainly on biomarkers and anthropometric parameters.

Regarding biomarkers, adiponectin, leptin, uric acid, high-sensitivity C-reactive protein, leptin, and Apoprotein-B showed good reliability as biomarkers in MetS screening in CCSs [5,23].

Regarding anthropometric parameters, the Body Mass Index (BMI) better estimates excess weight rather than excess fat and shows similar levels between pediatric brain cancer survivors and controls despite the higher adiposity in the former [24,25,26]

While the BMI underestimates obesity in survivors of pediatric blood cancer, increased adiposity can be assessed by using waist circumference, the waist-to-height ratio, or the tri-ponderal mass index [27,28,29].

To date, however, a tool able to predict body composition changes or detect them early and increased adiposity (and, therefore, increased likelihood of MetS onset) is still lacking, and few data are available regarding pediatric brain tumor survivors.

## 2. Materials and Methods

### 2.1. Objective of the Study

The primary endpoint of the study was as follows:

1. To analyze the differences in the results of bioelectrical impedance analysis (BIA) of body composition between male childhood brain tumor cancer survivors and healthy controls.

The secondary endpoint was as follows:

2. To evaluate the correlation between BIA results and the treatments performed (chemotherapy, radiotherapy, and steroid therapy).

### 2.2. Study Design and Inclusion and Exclusion Criteria

In this pilot, prospective, and observational study, 14 male childhood brain tumor survivors were compared to 14 healthy controls matched for sex and age. This group of patients was the same involved in a previous study of our group, and part of the results are the same as those reported in the same study (specifically the anthropometric and the treatment-related data) [30].

Cases were male brain cancer survivors attending their follow up at Pediatric Oncology Unit of Fondazione Policlinico Universitario “Agostino Gemelli” IRCCS in Rome in the period between June 2023 and September 2023. Inclusion criteria were as follows: diagnosis of brain cancer during first 16 years of life; be aged 12 years or older; have been subjected to cranial radiotherapy (with or without chemotherapy); disease remission for at least 5 years with a negative magnetic resonance in the last year; absence of congenital or acquired heart disease, strength disorders in the lower/upper limbs, or balance disorders; Karnofsky performance status > 90; and male sex. Exclusion criteria were follows: remission of disease for less than 5 years; age less than 12 years at the time of enrollment; and female sex.

Healthy controls were male volunteers attending visits in the Fondazione Policlinico Universitario “Agostino Gemelli” IRCCS Sport Medicine Unit matched for age. Inclusion criteria were follows: good physical condition without comorbidity; no history of cancer or lifelong history of steroid therapy longer than 7 consecutive days; and male sex.

Informed consent was obtained from the parents or the legal guardians of the enrolled patients or from patients for adults. The study was carried out following the Helsinki Declaration of Human Rights and was approved by the ethics committee of Fondazione Policlinico Universitario “Agostino Gemelli” IRCCS (protocol ID 5729, approval letter number 0019314/23 dated 20 June 2023).

Only male patients were included in this study in order to rule out misleading body composition changes due to menstrual cycle hormones.

### 2.3. Disease- and Treatment-Related Data

The following data were collected for each case:

1. Anthropometric parameters at the time of diagnosis: age, weight, height, BMI, BMI percentile, cancer histology, site of disease, and presence of metastasis.
2. Data related to the treatment: chemotherapy protocol, site and dose of radiotherapy, high dose of chemotherapy followed by autologous transplantation (ASCT), and duration of supportive steroid therapy during treatment.
3. Presence of endocrinological disfunction occurred during or after the end of oncological treatment.

### 2.4. Anthropometric Characteristics

For each participant (case and control), the following data were collected through physical examination:

1. Height, height-for-age percentile, and Z-score, calculated with a CDC growth charts-based percentile calculator available online (https://peditools.org/, last accessed on 19 February 2024).
2. Weight (measured in the absence of clothing except undergarments with electronic scales), weight-for-age percentile, and Z-score, calculated with a CDC growth chart-based percentile calculator available online (https://peditools.org/, last accessed on 19 February 2024).
3. BMI, calculated as (weight in kg)/(height in m)^2^, BMI-for-age percentile, and Z-score, calculated with a CDC growth chart-based percentile calculator available online (https://peditools.org/, last accessed on 19 February 2024).
4. Neck, chest, arm, wrist, thigh, and calf circumference in cm; waist circumference (reported in cm; it was measured as the circumference in the smallest point between the last rib and the top of the iliac crest); and hip circumference (reported in cm; it was measured at the major circumference point at the posterior extension of the buttocks).
5. Waist-to-hip ratio (WHR), expressed as the value obtained using the formula (waist circumference in cm)/(hip circumference in cm).
6. Waist-to-height ratio (WHtR), expressed as the value obtained using the formula (waist circumference in cm)/(height in cm), with a value of WHtR > 0.5 considered indicative of central obesity.

### 2.5. Body Composition Measurements

All subjects underwent body composition assessment, which was performed using a bioimpedance analyzer. Anthropometric parameters were also collected during the physical examination.

The impedance measurements were performed with a phase sensitive single frequency analyzer (BIA 101 AKERN Akern srl, Florence, Italy), which applies an alternating current of 800 µA at the frequency of 50 kHz (Akern s.r.l., Florence, Italy), and measurements were made following a standardized protocol according to the widely accepted methodology [31].

Participants were measured using tetrapolar configuration as described by Lukaski [32]: data were collected after 10 min of stationary supine position, with arms abducted 30° from the body and with a leg opening of 45°. After an accurate skin cleaning made rubbing hydroalcoholic solution, two adhesive electrocardiographic electrodes were placed on the dorsum of the wrist and two on back of the ankle on the right side of the body, with a distance of 5 cm between each other [33]. All patients were instructed to remove jewelry or metal accessories, maintain proper hydration, avoid strenuous physical exercise the day before, and arrive for the examination in a fasting state and with empty bladder. The evaluation room temperature was monitored and constantly maintained at around 23–24 °C [34].

The raw data of resistance (Rz), reactance (Xc), and phase angle (PA) were recorded once a stable condition was reached. Derived parameters were obtained from Rz and Xc using Bodygram^®®^ dashboard and its equations (Akern Srl, Firenze, Italy), as recommended by the producer of BIA 10142.

**Figure 1:**
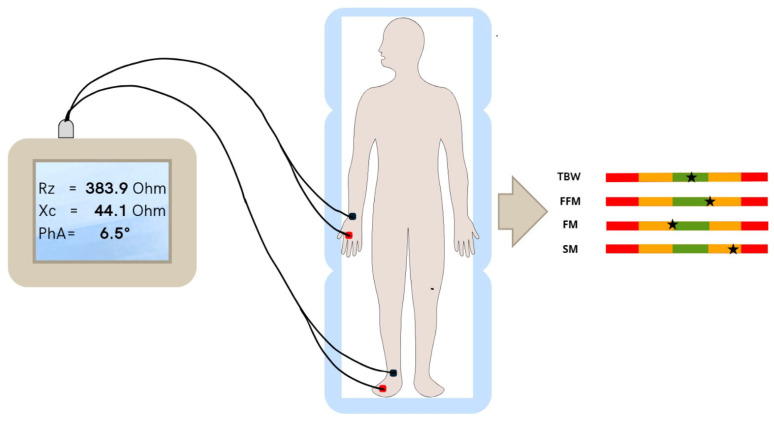
The methodology for the bioelectrical impedance analysis: the patient is positioned correctly on the examination bed and connected to the analysis apparatus via electrodes and a tetrapolar cable. The device then collects the raw data of resistance (Rz), reactance (Xc), and phase angle (PhA), which are subsequently analyzed and converted into the main body composition assessment parameters (e.g., fat mass, fat-free mass, and total body water) by a specific software (Bodygram Dashboard, Akern Srl, Firenze, Italy).

Derived parameters analyzed were the body cell mass (BCM), body cell mass index (BCMI, calculated as kilograms divided by squared meters), total body water (TBW), total body water expressed as percentage of body weight (TBW%), basal metabolic rate (BMR), extracellular water (ECW), extracellular water expressed as percentage of body weight (ECW%) fat mass (FM), fat mass expressed as percentage of body weight (FM%) fat mass index (FMI, calculated as kilograms divided by squared meters), fat-free mass (FFM), fat-free mass expressed as a percentage of body weight (FFM%) fat-free mass index (FFMI, calculated as kilograms divided by squared meters), skeletal muscle mass (SM), skeletal muscle mass index (SMI, calculated as kilograms divided by meters squared), appendicular skeletal muscle mass (ASMM), and standardized phase angle (SPA) (Figure 1).

### 2.6. Sample Size and Statistical Analysis

In this pilot study, we set the sample size at n = 14 patients compared to 14 controls. This number intercepts various expected proportions with a confidence level of 95% and a margin of error ranging from 5.21% (expected proportion of 1%) to 26.19% (expected proportion of 50%). This sample size is able to identify mean value differences between cases and controls in terms of effect size, equal to 0.98, with a power of 80% at a significance level of 5% (calculated with G*Power 3.1.9.7. software, Düsseldorf, Germany). Descriptive statistical analysis was performed for every parameter in the case and control groups. Levene’s test was used to assess homogeneity of variance, and Shapiro–Wilk’s test was used to assess normality of data distribution. Normally distributed continuous variables were compared with the Student’s *t*-test while the Mann–Whitney test was used for non-normally distributed variables or ordinal variables. Kendall’s test was used to assess the correlation between statistically significant variables for the cases and treatment-related data.

## 3. Results

### 3.1. Disease- and Treatment-Related Data

Fourteen male survivors of pediatric brain cancer participated in this exploratory, pilot, prospective, observational study and were compared to an equal number of healthy age- and sex-matched controls.

Table 1 shows the main demographic and anthropometric characteristics of the patients enrolled at the time of diagnosis.

At the time of diagnosis, the mean age of patients was 9.6 years (SD 5), the mean weight was 43.8 kg (SD 22.3), the mean weight percentile was 71.8 (SD 33.9), the mean height was 1.4 m (SD 0.3), and the mean height percentile was 52.8 (SD 33.3). The mean BMI was 21.1 (SD 4.8), and the mean BMI percentile was 87.9 (SD 17.3).

Table 2 shows the main clinical features related to the disease (histology and primary localization).

Regarding the disease data, a germ-cell tumor was diagnosed in eight patients (57%), a medulloblastoma in four (29%), and an ependymoma in two patients (14%). Eight patients (57%) had a tumor localized in the middle cranial fossa, and six (43%) had a tumor localized in the posterior cranial fossa. One patient, affected by a pineal germinoma, had a dorsal lumbar spinal cord metastasis.

Table 3 shows treatment-related data.

All patients were treated with cranial radiotherapy, and twelve of them (86%) were also treated with chemotherapy. The drugs used were etoposide, ifosfamide, cisplatin, carboplatin, vincristine, and lomustine, combined in different protocols.

Two patients (14%) (one affected by a germ-cell tumor of the pineal gland and the third ventricle and the other one affected by classic medulloblastoma) were also treated with high-dose chemotherapy, followed by an autologous stem cell transplant (ASCT), performed with the following drugs: thiotepa, busulfan, and cyclophosphamide.

Nine (64%) of the patients had endocrinological deficiencies at the time of enrollment, represented by panhypopituitarism (four patients, 29%), hypothyroidism (two patients, 14%), and GH deficiency (three patients, 21%).

Among the four patients affected by panhypopituitarism, two were affected by a pineal region tumor treated with local radiotherapy (one is in treatment with levothyroxine and desmopressin, the other with levothyroxine and hydrocortisone), another one was affected by a diencephalic and pituitary germ-cell neoplasia, treated with local radiotherapy and later with a substitutive therapy based on levothyroxine, hydrocortisone, testosterone, and desmopressin, while the last one had a posterior cranial fossa treated with a whole-brain radiotherapy, followed by focal and spinal boosts and substitutive therapy based on levothyroxine while refusing GH therapy.

The ones with hypothyroidism were two patients affected by a posterior cranial fossa tumor treated with whole-brain radiotherapy and boosts on focal and spinal lesions; they were in treatment with levothyroxine.

Two out of the three patients with GH deficiency had posterior cranial fossa cancer (ependymoma and medulloblastoma), while the other one had germ-cell cancer localized in the pineal region. They all received localized radiotherapy and later substitutive therapy with GH hormone.

At the time of enrollment, the mean age of patients was 25 years. The mean of the follow up was 171 months, 14.25 years from the end of treatment.

No relevant differences were found for education and/or occupation, disease duration, and socio-demographic characteristics.

### 3.2. Anthropometric Characteristics

Ten (71%) patients and nine (64%) healthy controls had a family history positive for diabetes, two patients (14%) and five (36%) controls had a familiar history of obesity, and ten (71%) patients and four (29%) controls had a family history positive for cardiovascular disease. No control subject smoked. Two patients (14%) smoked 1–2 cigarettes/day. The controls were also taller than the cases (*p* value = 0.015), with a higher percentile by age (*p* value = 0.007), and they also scored a higher weight (*p* value = 0.025) and a higher percentile by age (*p* value = 0.015).

There were no statistically significant differences between the cases and controls in terms of the BMI and waist, neck, chest, arm, hip, thigh, and calf circumferences. However, the WHtR was higher in cases than in controls, while mean WHR values were higher in controls than in cases. Additionally, five (36%) of cases and three (21%) of controls exhibited a pathological WHtR (>0.5). Table 4 presents a comparison of the anthropometric characteristics of the patients and controls at the time of enrollment.

### 3.3. BIA Results

Pediatric brain cancer survivors showed statistically significantly lower mean values in terms of the basal metabolic rate (BMR, *p*-value = 0.004), body cell mass (BCM, *p*-value = 0.003), body cell mass index (BCMI, *p*-value = 0.045), fat-free mass (FFM, *p*-value = 0.004), skeletal muscle mass (SM, *p*-value = 0.007), skeletal muscle mass index (SMI, *p*-value = 0.035), and appendicular skeletal muscle mass (ASMM, *p*-value = 0.002). Cases also showed a statistically significantly higher mean value of resistance (RZ, *p*-value = 0.018) when compared to the controls. Table 5 shows the results of the comparison between the two groups.

### 3.4. Correlation Between Treatment Performed and BIA Parameters in the Survivors

In the correlation analysis, we found a significant inverse correlation between BIA parameters and the treatments administered. Specifically, we observed that the BMR, BCM, FFM, and ASMM were all significantly inversely correlated to the total dose of Carboplatin, with strong statistical evidence (tau = −0.601, *p*-value = 0.018), (tau = −0.599, *p*-value = 0.025), (tau = −0.601, *p*-value = 0.018), and (*p*-value = 0.045), respectively.

The table showing the relationships between BIA parameters and the treatments administered is reported in Supplementary Materials (Table S1).

**Figure 2:**
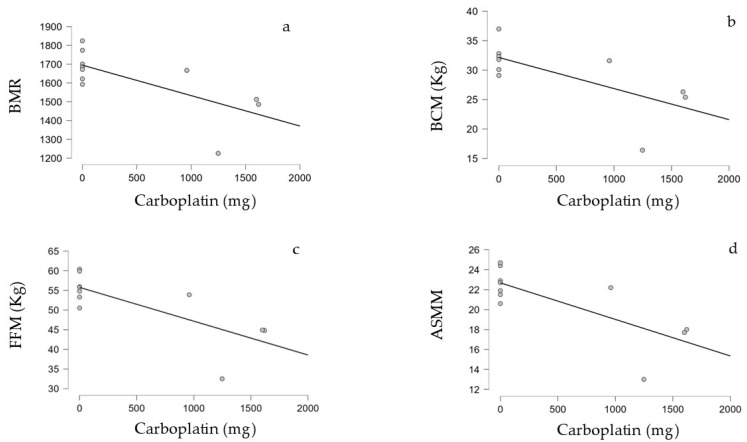
Graphic representations of the significant correlations: (**a**) the correlation between carboplatin in mg and the BMR; (**b**) the correlation between carboplatin in mg and the BCM in kg; (**c**) correlation between carboplatin in mg and the FFM in kg; (**d**) the correlation between carboplatin in mg and the ASMM.

Table 6 shows only the above-mentioned significant correlations. Figure 2 shows the graphic representations of the significant correlations.

## 4. Discussion

Nowadays, pediatric brain tumors are characterized by an overall survival rate of roughly 80%. CCSs, however, develop several treatment-related complications. Among these, overweight, adiposity, and obesity play a key role in the pathogenesis of MetS, a major risk factor for cardiovascular death.

In this prospective observational study, we enrolled 14 male childhood brain tumor survivors and compared their anthropometric parameters to 14 age- and sex-matched healthy controls. We found out that the control group scored a higher weight value and a higher weight percentile by age; the controls were taller than the cases, with a higher height percentile by age, although there was no significant difference in terms of the BMI and waist, neck, chest, arm, hip, thigh, and calf circumferences between the two groups. Furthermore, a pathological WHtR (>0.5) was observed in five (36%) cases and three (21%) controls. The cases had a high WHtR than the controls, while mean WHR values were higher in the controls than in the cases.

This result agrees with two reviews published by Wang: CCSs show increased total and central adiposity despite similar BMI values when compared to healthy controls [24,25].

Furthermore, Blijdorp et al. demonstrated that the BMI and WHR are not reliable indexes when assessing obesity in adults who survived a pediatric central nervous system tumor, and their use can lead to underestimation [29]. The WHtR (with a cut-off value of 0.5 in several adult populations), on the other hand, is a good predictor of metabolic risk, better correlates with visceral fat, and has been associated with cardiovascular mortality. Unfortunately, few data are available for children, and there is not a universally accepted cut-off [26,28].

In order to better investigate the difference between the cases and controls in terms of an early MetS predictor, we performed a BIA. CCSs had lower mean values in terms of the basal metabolic rate (BMR), body cell mass (BCM), body cell mass index (BCMI), fat-free mass (FFM), skeletal muscle mass (SM), skeletal muscle mass index (SMI), and appendicular skeletal muscle mass (ASMM). The cases also showed a statistically significantly higher mean value of resistance (RZ) when compared to the controls.

Specifically, the BCM is the metabolically active component of the FFM and is the single best predictor of malnutrition, while the BCMI (BCM/squared height) is a more sensitive parameter than the BMI when assessing a subject’s muscle mass and tissutal protein status [35,36,37,38].

These findings are coherent with a decrease in skeletal muscle mass, which is a key component of the definition of sarcopenia: although there is not a universally accepted definition of sarcopenia, the most widely used one is the EWGSOP2 definition, based on the following three criteria: low muscle strength, low muscle quality or quantity, and low physical performance (only criterion 1 means probable sarcopenia, the addition of criterion 2 is diagnostic for sarcopenia, and the three criteria altogether define severe sarcopenia). This study shows only the presence of criterion 2 among pediatric brain tumor survivors, but a previous study performed on the same court reported lower cardio-pulmonary exercise test scores in the cases when compared to the controls, reflecting a lower level of physical performance (despite a similar level of physical activity) in the case group [30].

Sarcopenia (clinically or radiologically defined) is a well-known and widely reported late effect of cancer treatment and has been documented for leukemia [39,40,41], Ewing sarcoma [42], Wilms tumors [43], hepatoblastomas [44], neuroblastomas [45,46,47,48], and bone and soft tissues sarcomas [49], but few data are available for pediatric brain tumors and in pediatric brain tumor survivors, which is a large but still poorly understood population; our work therefore highlights the potential for cancer-related sarcopenia in this type of patient as well, an aspect that is certainly relevant and worthy of further investigation. Indeed, what we have detected is only the loss of muscle mass, but the proof that this parameter is associated with low muscle strength is mandatory in the EWGSOP2 definition of sarcopenia. It is known, in fact, that children with brain cancer are the most prone to develop overweight and obesity and that they show a lower fat-free mass than children with hematologic malignancies [50,51]; this highlights that the correct reading perspective of this condition should probably be sarcopenic obesity, where both obesity and sarcopenia contribute to the development of MetS. Various evidence from other clinical settings pointed out that high cytokine levels can affect protein metabolism both directly, via its effect on muscle amino acid balance [52,53], and indirectly, via insulin sensitivity, affecting peripheral insulin sensitivity [54]. A loss of lean mass, obesity, and inflammatory status may thus indicate the same phenomenon, where the final effect is not only the onset of complications related to the metabolic syndrome but also a significant worsening of overall quality of life and overall survival [55].

Thus, a detailed assessment of body composition makes it possible to investigate the nutritional status of childhood brain tumor survivors even more thoroughly and to analyze the role played by each component (e.g., lean mass, fat mass) in the development of relevant clinical disorders (such as but not limited to MetS).

It is important to note that a BIA is a straightforward, non-invasive, and cost-effective method of analysis. However, its diagnostic accuracy is not optimal. The data obtained through this method require further investigation and confirmation with more reliable techniques, such as dual-energy X-ray absorptiometry and an ultrasound analysis of visceral and subcutaneous fat tissue. Additionally, the results should be validated using gold standards, such as computed tomography and magnetic resonance imaging. Further studies in this direction represent an intriguing avenue for further investigation.

We have also observed an inverse correlation between the total dose of carboplatin and the values of the BMR, BCM, FFM, and ASMM. As the carboplatin dose increased, the reported scores for the BMR, BCM, FFM, and ASMM decreased. Interestingly, we did not find this correlation between these parameters and the dose of cranial radiotherapy, duration of steroid therapy, and total dose of cisplatin, ifosfamide, and cyclophosphamide.

There is currently no causal correlation reported in the literature between sarcopenia or adiposity in pediatric brain tumor survivors and carboplatin. Carboplatin is a second-generation platinum compound known to be less toxic and more tolerable than cisplatin [56], making it the most widely used chemotherapy drug for pediatric brain tumors. Although this may be an accidental finding due to our limited sample size, further studies are necessary to thoroughly investigate this topic and ensure its validity.

## 5. Conclusions

A BIA showed promising results in detecting adiposity and body composition changes that predispose pediatric brain tumor survivors to development of MetS and sarcopenia early and needs to be considered as a screening tool in this population, especially in those receiving high doses of carboplatin.

## Figures and Tables

**Table 1:** Patient-related data (n = 14).

	Number (%) or Mean (SD)
Age at diagnosis (years)	9.6 (5)
Weight (kg)	43.8 (22.3)
Weight percentile	71.8 (33.9)
Weight Z-score	0.9 (1.2)
Height (m)	1.4 (0.3)
Height percentile	52.8 (33.3)
Height Z-score	0.1 (1.2)
BMI (kg/sqcm)	21.1 (4.8)
BMI percentile	87.9 (17.3)
Age at the enrollment (years)	24.9 (3.9)
Time of follow up at the enrollment (months)	171 (54)

**Table 2:** Disease-related data (n = 14).

	Number of Patients (%)
**Histology**	
Germ-cell tumor	8 (57%)
Medulloblastoma	4 (29%)
Ependymoma	2 (14%)
**Primary localization**	
Anterior cranial fossa	0 (0%)
Middle cranial fossa	8 (57%)
Posterior cranial fossa	6 (43%)

**Table 3:** Treatment-related data (n = 14).

	Number of Patients (%) or Mean (SD)
Patients subjected to cranial radiotherapy	14 (100%)
Patients subjected to spinal radiotherapy	5 (36%)
Cranial radiotherapy dose (Gy)	56.7 (16.6)
Spinal radiotherapy dose (Gy)	30 (0)
Patients subjected to chemotherapy	12 (86%)
Patients subjected to steroid therapy (more than 14 days)	10 (71%)
Patients subjected to ASCT *	2 (14%)
Relapsed disease	2 (14%)

* ASCT: autologous stem cells transplant.

**Table 4:** A comparison of anthropometric characteristics of the patients and controls at the time of enrollment.

	Cases	Controls	*p* Value
	[Mean (SD) or Number of Patients (%)]	[Mean (SD) or Number of Patients (%)]	
Age (years)	24.93 (3.89)	24.64 (2.92)	0.834
Weight (kg)	67.89 (10.89)	76.81 (8.85)	0.025
Weight percentile	48.23 (19.08)	64.79 (23.91)	0.015 *
Weight Z-score	−0.31 (1.12)	0.41 (0.70)	0.347
Height (m)	1.68 (0.10)	1.77 (0.08)	0.015 *
Height percentile	28.67 (19.65)	51.79 (33.23)	0.007 *
Height Z-score	−1.19 (1.40)	0.06 (1.13)	0.434
BMI (kg/m^2^)	23.99 (3.22)	24.59 (3.19)	0.624
BMI percentile	57.34 (26.90)	58.91 (26.29)	0.877
BMI Z-Score	0.11 (1.12)	0.31 (0.83)	0.607
Waist circumference (cm)	83.04 (8.31)	80.36 (9.83)	0.079
Hip circumference (cm)	91.07 (7.44)	87.86 (12.08)	0.178
WHR	0.91 (0.05)	0.92 (0.07)	<0.002 *
WHtR	0.49 (0.05)	0.45 (0.06)	0.049 *
WHtR > 0.5	5 (36%)	3 (21%)	0.402
Neck circumference (cm)	36,214 (3.63)	37,900 (1921)	62
Chest circumference (cm)	94,500 (8644)	91,846 (13,266)	802
Wrist circumference (cm)	17,057 (1588)	17,069 (781)	554
Arm circumference (cm)	29,500 (3627)	29,885 (2952)	971
Thigh circumference (cm)	54,857 (14,347)	53,808 (4381)	388
Calf circumference (cm)	36,129 (3879)	37,192 (2411)	413

* Statistically significant *p* < 0.05

**Table 5:** A comparison of BIA parameters between the patients and controls at the time of enrollment.

	Cases	Controls	*p*-Value
	[Mean (SD) or 50th Percentile (IQR)]	[Mean (SD) or 50th Percentile (IQR)]	
BSA	1.804 (0.214)	1.901 (0.126)	0.158
BMI	23.679 (3.715)	24.900 (2.476)	0.316
XC	68.273 (12.650)	63.615 (6.764)	0.262
PA	6.882 (0.946)	7.438 (0.690)	0.11
BMR	1614.636 (163.399)	1828.177 (159.454)	0.004 *
BCM (kg)	29.280 (5.611)	37.185 (5.498)	0.003 *
BCMI (kg/m^2^)	10.755 (1.545)	11.900 (1.087)	0.045 *
FFM (kg)	51.518 (8.111)	61.677 (7.179)	0.004 *
FFM (%)	80.064 (5.285)	79.908 (9.186)	0.961
FFMI (kg/m^2^)	18.609 (1.795)	19.785 (1.442)	0.089
FM (kg)	13.027 (3.966)	14.554 (5.462)	0.477
FMI (kg/m^2^)	4.745 (1.705)	4.762 (2.087)	0.984
FM (%)	19.936 (5.285)	18.323 (6.139)	0.502
TBW (L)	34.818 (6.938)	44.869 (5.422)	<0.001 *
TBW (%)	56.727 (5.603)	59.069 (4.638)	0.247
SPA	0.073 (1.210)	0.798 (1.005)	0.122
SM (kg)	28.100 (3.714)	33.108 (4.162)	0.007 *
SMI	9.873 (0.852)	10.608 (0.754)	0.035 *
ASMM	20.873 (3.437)	25.592 (3.241)	0.002 *
Rz	527.00 (91.00)	491.00 (56.00)	0.018 *
Hydration	72.700 (3.650)	72.900 (0.475)	0.084
ECW (%)	41.3	40.00 (1.800)	0.118
ECW (L)	39.664 (2.842)	39.985 (1.871)	0.017 *

* Statistically significant *p* < 0.05. BSA: body surface area, BMI: body mass index, BMR: basal metabolic rate, XC: reactance, BCM: body cell mass, BCMI: body cell mass index, FM: fat mass, SPA: standardized phase angle, ASMM: appendicular skeletal muscle mass, SMI: skeletal muscle mass index, SM: skeletal muscle mass, FFMI: fat-free mass index, FFM: fat-free mass, FFM (%): fat-free mass as percentage of body weight; FMI: fat mass index, FM (%): fat mass as percentage of body weight; ECW: extracellular body water, ECW (%): extracellular body water as percentage of body weight, TBW: total body water, TBW (%): total body water as percentage of body weight, PA: phase angle, Rz: reactance. Rz, Hydration, TBW (%), FFM (%), ECW(%), FM (%) are expressed as 50th percentile (IQR: inter-quartile range), and their *p*-values are relative to Mann–Whitney Test.

**Table 6:** Significant correlations between total dose of Carboplatin and BIA parameters.

		Carboplatin (Total Dose in mg)
BMR	Kendall’s Tau	−0.601
	*p*-value	0.018
BCM (kg)	Kendall’s Tau	−0.599
	*p*-value	0.025
FFM (kg)	Kendall’s Tau	−0.601
	*p*-value	0.018
ASMM	Kendall’s Tau	−0.509
	*p*-value	0.045

BMR: basal metabolic rate, BCM: body cell mass; FFM: fat-free mass, ASMM: appendicular skeletal muscle mass.

## Data Availability

Data are not available due to privacy restriction.

## Supplementary Materials

The following supporting information can be downloaded at https://www.mdpi.com/article/10.3390/diseases12120306/s1, Table S1 shows the relationships between BIA parameters and the treatments administered.
