# Supplementary material for: Bioelectrical Impedance Analysis of Body Composition in Male Childhood Brain Tumor Survivors
Source: Diseases. 2024 Nov 28;12(12):306. doi: 10.3390/diseases12120306 (PMC11727618; doi:10.3390/diseases12120306)
Supplement: Supplementary file 1 [file diseases-12-00306-s001.zip › diseases-3288314-supplementary.pdf]

Table S1 shows the relationships between BIA parameters and the treatments administered

		Cranial Radiotherapy (Gy)	Steroid therapy (months)	Carboplatin (total dose in mg)	Cisplatin (total dose in mg)	Ifosfamide (total dose in mg)	Cyclophosphamide (total dose in mg)
BMR	Kendall's Tau	-0.109	-0.277	-0.601	0.128	0.285	0.000
	p-value	0.708	0.275	0.018	0.608	0.271	1.000
BCM (kg)	Kendall's Tau	0.098	-0.218	-0.599	-0.054	0.181	0.109
	p-value	0.761	0.416	0.025	0.839	0.517	0.684
BCMI (kg/m2)	Kendall's Tau	-0.074	-0.420	-0.210	0.086	0.026	-0.210
	p-value	0.802	0.100	0.411	0.732	0.920	0.411
FFM (kg)	Kendall's Tau	0.109	-0.324	-0.601	0.085	0.234	0.139
	p-value	0.708	0.202	0.018	0.732	0.367	0.585
TBW (L)	Kendall's Tau	0.109	-0.092	-0.416	-0.256	-0.078	0.324
	p-value	0.708	0.716	0.101	0.305	0.764	0.202
ASMM	Kendall's Tau	-0.109	-0.277	-0.509	0.085	0.285	0.185
	p-value	0.708	0.275	0.045	0.732	0.271	0.466
ECW (L)	Kendall's Tau	0.222	-0.023	-0.490	0.151	0.236	0.140
	p-value	0.451	0.927	0.055	0.548	0.366	0.584
RZ	Kendall's Tau	-0.109	0.139	0.462	0.000	-0.078	-0.092
	p-value	0.708	0.585	0.069	1.000	0.764	0.716
